# The Mediterranean Diet Benefit on Cardiovascular Hemodynamics and Erectile Function in Chronic Heart Failure Male Patients by Decoding Central and Peripheral Vessel Rheology

**DOI:** 10.3390/nu13010108

**Published:** 2020-12-30

**Authors:** Athanasios Angelis, Christina Chrysohoou, Evangelia Tzorovili, Aggeliki Laina, Panagiotis Xydis, Ioannis Terzis, Nikos Ioakeimidis, Konstantinos Aznaouridis, Charalambos Vlachopoulos, Konstantinos Tsioufis

**Affiliations:** First Cardiology Clinic, Hippokration Hospital, School of Medicine, University of Athens, 11527 Athens, Greece; nasosangelis@gmail.com (A.A.); evangeliatzo@gmail.com (E.T.); agelikilaina@hotmail.com (A.L.); panosxydis@yahoo.gr (P.X.); ioannisdterzis@gmail.com (I.T.); nioakim@gmail.com (N.I.); conazna@yahoo.com (K.A.); cvlachop@otenet.gr (C.V.); ktsioufis@gmail.com (K.T.)

**Keywords:** mediterranean diet, prolactin, chronic heart failure, erectile function

## Abstract

Background: Mediterranean diet was evaluated on erectile performance and cardiovascular hemodynamics, in chronic heart failure patients. Methods: 150 male stable heart failure patients were enrolled in the study (62 ± 10 years, New York Heart Association (NYHA) classes I–II, ejection fraction ≤40%). A detailed echocardiographic evaluation including estimation of the global longitudinal strain of the left ventricle and the systolic tissue doppler velocity of the tricuspid annulus was performed. Erectile dysfunction severity was assessed by the Sexual Health Inventory for Men-5 (SHIM-5) score. Adherence to the Mediterranean diet was evaluated by the MedDietScore. Results: The SHIM-5 score was positively correlated with the MedDietScore (*p* = 0.006) and augmentation index (*p* = 0.031) and inversely correlated with age (*p* = 0.002). MedDietScore was negatively associated with intima-media-thickness (*p* < 0.001) and serum prolactin levels (*p* = 0.05). Multi-adjusted analysis revealed that the inverse relation of SHIM-5 and prolactin levels remained significant only among patients with low adherence to the Mediterranean diet (*p* = 0.012). Conclusion: Consumption of Mediterranean diet benefits cardiovascular hemodynamics, while suppressing serum prolactin levels. Such physiology may enhance erectile ability independently of the of the left ventricle ejection fraction.

## 1. Introduction

The prevalence of chronic heart failure (CHF) is estimated around 2% of the adult population, mainly older aged men [1]. CHF causes substantial morbidity, influences quality of life and reduces life expectancy. Moreover, coronary artery disease accounts for two-thirds of CHF cases, in developed countries. Central hemodynamics and particularly wave reflection amplification constitutes a vital parameter of CHF physiology. Increased arterial stiffness influences the cardiovascular mechanisms resulting in adverse cardiovascular outcomes and poor quality of life, including sexual performance. Erectile dysfunction (ED) is strongly correlated with sex-hormones and prolactin levels, and has been used as a proxy of quality of life. ED has been related with hemodynamic status and seems to display peripheral vessel rheology, even in patients with atherosclerosis and CHF, while the role of serum prolactin role is still debated in CHF mechanisms is still under consideration [2,3,4,5,6,7,8,9].

Dietary habits are recognized as important and modifiable risk factors of cardiovascular disease (CVD), not only in primary, but also in secondary prevention [1,10]. Since the 1970s, large-scale community-based interventions revealed that favorable dietary changes, as a part of an integrated and multi-dimensional lifestyle approach, have led to notable reduction in CVD morbidity and mortality [11]. The current trend in nutrition-based epidemiology is to examine the diet-health interaction through a holistic approach, instead of focusing on isolated nutrients and food items. Several dietary patterns have been studied in the past 2–3 decades for their effect on CVD risk. Among them, the Mediterranean diet is one of the most well studied dietary patters regarding its influence on CVD risk. Mediterranean diet is characterized by high consumption of monounsaturated fatty acids, mainly from olives and olive oil, and suggests daily consumption of fruits, vegetables, whole grain cereals and low-fat dairy products, weekly consumption of fish, poultry, tree nuts and legumes, a relatively low consumption of red meat, and moderate daily consumption of alcohol, usually with meals. It is a different dietary pattern as compared to other healthy patterns, like the ketogenic diet which is low in carbohydrates (lower than the Mediterranean diet), but high in fat (higher that Mediterranean diet). According to the 2010 definition of the United Nations Educational, Scientific and Cultural Organization (UNESCO), the Mediterranean diet (from the Greek word diaita, which means way of life), constitutes “a set of skills, knowledge, practices and traditions ranging from the landscape to the table, including the crops, harvesting, fishing, conservation, processing, preparation and, particularly, consumption of food”. Several clinical trials and epidemiological studies have already showed that the Mediterranean diet’s health benefits stem from its anti-inflammatory and vasoprotective properties [10,12,13]. However, the impact of the Mediterranean diet on CVD risk in patients with CHF, and particularly its influence on cardiovascular hemodynamics, has been poorly studied and understood.

The scope of this work was to examine the role of Mediterranean diet on erectile function and cardiovascular hemodynamics, in patients with chronic heart failure, in order to illustrate central and peripheral vessel rheology and prolactin physiology.

## 2. Materials and Methods

### 2.1. Design and Setting

This is an epidemiological study that was conducted at the First Cardiology Clinic of Hippokration General Hospital of Athens, in Greece, during 2019–2020.

### 2.2. Sample

We enrolled 157 clinically stable CHF, male patients (62 ± 10 years) who consecutively visited the outpatient unit of our hospital. Eligibility criteria included age of at least 18 years, with left ventricular ejection fraction of ≤40% and symptoms according to New York Heart Association (NYHA) class II or higher symptoms. Patients were receiving standard medical therapy on an optimal tolerated dose, including an angiotensin-converting enzyme inhibitor or angiotensin-receptor blocker or a sacubitril-valsartan combination, a mineralocorticoid receptor antagonist plus a beta-blocker and diuretic. If indicated, they were treated with appropriate device therapy (i.e., ICD—Implanted cardiac defibrillator). Newly (i.e., within 1 month) diagnosed heart failure patients were excluded, as well individuals with cancer, active infection or other major systemic disease.

### 2.3. Bioethics

The study conformed to the principles of the Declaration of Helsinki and was approved by the Ethics Committee of Cardiology Clinic of the Hippokration Hospital (Institutional Review Board 0001/10/6/2019). All subjects had signed a written informed consent form.

### 2.4. Dietary Assessment and Evaluation of Adherence to the Mediterranean Diet

Nutritional habits assessment was based on a validated semi-quantitative food-frequency questionnaire [13]. All participants were asked to report the average weekly intake of several foods and beverages they consume; the MedDietScore was used to evaluate the adherence to the Mediterranean dietary pattern [13]. MedDietScore is an 11-item index with questions regarding the frequency of consumption of the main nutrients that characterize the Mediterranean Diet (MedDiet). Individual ratings (from 0 to 5 or the reverse) were assigned in each of the 11 items according to their position in the Mediterranean diet pyramid. For the consumption of items presumed to be close to this pattern (i.e., consumption on a daily basis or more than 18 servings/month of non-refined cereals, fruits, vegetables, legumes, olive oil, fish, and potatoes) a score of 5 was given; the contrary score 0 was given when someone reported no consumption, score 1 when they reported consumption of 1–4 servings/month, score 2 for 5–8 servings/month, score 3 for 9–12 servings/month, and score 4 for 13–18 servings/month. For food habits presumed to be distinct from this dietary pattern (i.e., rare consumption of healthy foods or frequent intake of meat and meat products, poultry and full fat dairy products), scores were assigned on a reverse scale. Concerning alcohol drinking, score 5 was assigned for consumption of <300 mL of alcohol/day, score 0 for consumption of <700 mL/day and scores 4 to 1 for consumption of 300, 400–500, 600 and 700 or 0 mL/day respectively. Higher values of this diet score indicate greater adherence to the Mediterranean diet (theoretical range 0–55).

### 2.5. Measurements

Baseline evaluation included information about socio-demographic characteristics, history of hypertension, dyslipidemia and diabetes mellitus as well as dietary and other lifestyle habits (i.e., smoking status and physical activity). In particular, smokers were defined as those who were currently smoking at least 1 cigarette/day during the past year. Frequency and duration per time of the engagement in physical activities, e.g., organized sport clubs, free style, was recorded and physical fitness levels were calculated in metabolic equivalents per minute/day (METS). Body mass index (BMI) (kg/m^2^) was estimated by dividing measured weight (kg) by measured standing height squared (m^2^). Arterial blood pressure was measured in patients in a sitting position, calm for at least 30 min; those with average systolic/diastolic blood pressure levels ≥140/90 mmHg or who were under antihypertensive medication were classified as hypertensives. Hyperlipidemia was defined as a history of hypolipidemic treatment or and total blood cholesterol level ≥200 mg/dL. The presence of diabetes mellitus was defined as receiving hypoglycemic medication or having fasting glucose at least 126 mg/dL or HbA1c  > 6.5%. Serum prolactin and testosterone levels were measured by a radio-immuno-assay method. Patients were instructed to abstain from food and smoking for 12 h and blood samples were taken before 9:00 am.

### 2.6. Cardiac Ultrasound and Ergometric Parameters

Echocardiographic assessment was performed in all patients by using a GE 5500 series equipment with a multifrequency transducer (2.5–4 MHz) and tissue doppler imaging (i.e., TDI) technology. Left ventricular ejection fraction (EF) was calculated by the Simpson’s method, while right ventricular systolic function was evaluated upon the systolic component of the Tissue Doppler velocity of the tricuspid annulus (SRV). Left atrial (LA) maximal volume and global longitudinal strain of the left ventricle (GLPS) were measured (biplane modified Simpson method). In cardiorespiratory exercise (ergometric bicycle) VO2max (maximum oxygen consumption), VE/VCO2 (ratio of ventilation to carbon dioxide) and the METS (i.e., metabolic equivalents per min/day) achieved were evaluated. The ultra-sonographic examinations were carried out by a group of 4 clinicians who had at least 5-year experience in ultra-sonography in a clinical setting.

### 2.7. Vascular Measurements

Carotid intima-media thickness (IMT) measurements were performed with the patient lying in the supine position and with the neck rotated to the opposite side of the examination; it was measured in both right and left common carotid artery (1 cm proximally to the carotid bulb), the carotid bulb and the internal carotid artery (B-mode ultrasound imaging, 14.0-MHz multifrequency linear array probe, Vivid 7 Pro; General Electric Healthcare, Milwaukee, WI, USA). Three measurements of the maximal IMT in the far wall during diastole were averaged, and the summary was calculated for both carotid arteries. The presence of plaque was defined as a clearly identified area of focally increased IMT greater than 1.5 mm or 50% increase as compared to adjacent wall IMT [14,15]. Carotid-femoral pulse wave velocity (PWV) that evaluates central arterial stiffness was calculated by measuring pulse transit time and the distance travelled between 2 recording sites (carotid and femoral). A validated, non-invasive device (Complior^®^, Artech Medical, Pantin, France) allowing online pulse wave recording and automatic PWV calculation was used. Wave reflection amplification analysis was performed by a radial artery tonometry device (Sphygmocor System-Atcor Medical, Sydney, Australia) [14,15]. Peripheral pressure waveforms were recorded at the radial artery using a hand-held high-fidelity tonometer and calibrated by using arterial pressures measured at the brachial artery. The Augmentation Index (AIx), a measure of wave reflection amplification, was calculated as the ratio of the augmentation of aortic systolic pressure due to the reflected waves divided by the aortic pulse pressure and normalized for a heart rate of 75 bpm.

### 2.8. Evaluation of Erectile Dysfunction

Diagnosis and evaluation of the severity of erectile dysfunction were based on clinical symptoms, duration of the erectile disorder, and the score of the five-item form of the International Index of Erectile Function, the Sexual Health Inventory for Men (SHIM-5) score (less than 21 indicates erectile dysfunction). The lower the score the more severe the erectile pathology [16,17]. The vascular origin of the erectile disorder was based on the absence of any diagnosed psychological, neurologic or anatomic abnormalities, pelvic surgery or trauma, or history of malignancy and endocrine gland disorders.

### 2.9. Statistical Analysis

The working sample of *n* = 150 participants was found to be adequate, i.e., achieving 83% statistical power, to evaluate 1 standard deviation changes (two-sided hypotheses) on the tested hemodynamic markers by tertile change of the MedDietScore, at 5% significance level. Continuous variables are presented as mean values ± standard deviation. Categorical variables are presented as absolute values and relative frequencies. One-way analysis of variance (ANOVA) was used to evaluate associations between mean values of continuous variables between 3 or more independent groups. The normal distribution of the continuous variables was assessed using the Shapiro-Wilk test. The Kruskal-Wallis test was used as an alternative non-parametric approach in the case on non-normally distributed variables. Correlations between continuous variables were evaluated by the calculation of Pearson’s *r*-coefficient or the partial correlation coefficient, after adjusting for potential confounding factors. Linear regression models were estimated to assess the association of vascular measurements, SHIM-5 score, GLPS and hormone levels with adherence to the Mediterranean diet. Regression estimates are presented as standardized b-coefficients. Mediation analysis, using Baron and Kenny’s approach, was applied to evaluate the potential intervening role of the Mediterranean diet on the relationships between prolactin, testosterone levels and cardiac hemodynamic markers. All reported *p* values are based on two-sided tests. The SPSS statistical software package version 23.0 (Statistical Package for Social Sciences, SPSS Inc., Chicago, Illinois, USA) was used for all statistical calculations.

## 3. Results

The majority of the patients, i.e., 89%, were NYHA class II, and the rest 11% were class III. Mean EF was 30.5% (7.9) (according to the study’s protocol all patients had EF <40%), mean MedDietScore was 27.9 (3.4) (suggesting moderate adherence to the Mediterranean dietary pattern, i.e., 27.9 out of 55 theoretical total score = 50.7%), and mean body mass index (BMI) was 30.0 kg/m^2^ (8.7), whereas 45.8% were considered as obese (i.e., BMI >29.9 kg/m^2^). Demographic and clinical characteristics of the participants by level of adherence to the Mediterranean diet are presented in Table 1. Unadjusted analyses revealed an inverse association between the MedDietScore categories of adherence and prolactin levels (*p* = 0.05), IMT (*p* < 0.001), and a positive association with testosterone (*p* = 0.02) and SHIM-5 score (*p* = 0.006). No other significant associations between Mediterranean diet and cardiovascular hemodynamics markers were observed in the unadjusted analyses.

However, residual confounding may exist, therefore, multi-adjusted models were fitted having MedDietScore as independent factor and various hemodynamics markers as outcomes, adjusting for age, BMI, physical fitness levels of the participants and history of diabetes, hyperlipidemia and hypertension (Table 2). Data analysis revealed a positive association of the MedDietScore with SRV (*p* = 0.019) and the SHIM-5 index (*p* = 0.002), as well as an inverse association with AIx (*p* = 0.014), and IMT (*p* = 0.004). No significant associations were observed between MedDietScore with cardiac hemodynamic markers, as well as testosterone and prolactin levels (all *p*-values > 0.10).

Moreover, the impact of physical fitness on the hemodynamic variables examined was also evaluated; it was revealed that physical fitness was inversely associated with AIx (*p* = 0.010), and IMT (*p* = 0.01), after adjusting for age, BMI, history of diabetes, hyperlipidemia, hypertension, EF.

To further evaluate the role of sex-related hormones, i.e., testosterone and prolactin, on cardiac hemodynamics of CHF patients, multi adjusted correlation analyses were applied (Table 3). Prolactin was positively correlated with SRV (*p* < 0.001), with Aix (*p* = 0.03), and inversely correlated with VO_2_ at max (*p* = 0.03), as well as with SHIM-5 score (*p* = 0.01). Testosterone was inversely correlated with prolactin (*p* < 0.001), and positively with SHIM-5 score (*p* = 0.04). Furthermore, SHIM-5 score was inversely correlated with age (*r* = −0.677, *p* = 0.002), and positively correlated to AIx (*r* = 0.338, *p* = 0.031). To further evaluate the role of Mediterranean diet adherence on the relationships between sex-related hormones and cardiac hemodynamics, a mediation analysis was applied. In particular, when the level of adherence to the Mediterranean diet was taken into account prolactin levels were not associated anymore with SRV (*p* = 0.959), or VO_2_ max (*p* = 0.528). Moreover, prolactin levels were associated with reduced SHIM-5 score only among participants with low adherence to the Mediterranean diet (i.e., lowest tertile, b-coefficient: −0.559, *p* = 0.012), whereas among the other higher adherence groups no significant association between prolactin and erectile function was observed. In addition, adherence to the Mediterranean diet seemed to play a mediating role in the relationship between testosterone levels and erectile function. In particular, among participants who reported low adherence, testosterone levels were not associated with SHIM-5 score; however, among those who reported good or very good adherence (i.e., 2nd and 3rd tertile), testosterone levels were positively associated with SHIM-5 score (*p*-values <0.01).

## 4. Discussion

The present study revealed a multifaceted beneficial impact of the Mediterranean diet in middle-aged men with CHF, by ameliorating central hemodynamics, right ventricular function and deescalating parameters of arterial stiffness, while exerting atheroprotective effect with suppressed circulating serum prolactin levels. Such physiology enhances erectile performance, an important component of quality of life in the male population with heart failure.

Several studies underlined the anti-inflammatory properties of the MedDiet and its components and the positive impact on primary and secondary cardiovascular prevention [13,18,19,20]. In heart failure patients, higher adherence to the MedDiet has been associated with reduced NT-proBNP (NT-pro Brain Natriuretic Peptide levels), oxidized low-density lipoprotein and Lp(a) levels [21] while it seems to reduce the risk of a heart failure incident, as shown in a population-based cohort of 32,921 subjects [22]. Concerning the relationship between the MedDiet and left ventricular dysfunction, we revealed in a previous study of 372 heart failure patients that higher adherence to this dietary model ameliorates left ventricle diastolic function and, in particular, the ventricular filling physiology (left atrium ejection fraction and E/A ratio) [19]. Another study reported that NYHA class symptoms negatively correlate with the adherence to the MedDiet pointing to a modulating role in the severity of clinical presentation [23].

According to previous studies, in patients with systolic heart failure, the presence of right ventricular dysfunction is frequent and has been related to increased mortality. In our study, right ventricular function, as it was evaluated by tissue Doppler imaging, was positively related to MedDiet linking higher adherence to a healthy diet with amelioration of the right ventricular systolic function. Interestingly, the favorable effect of the MedDiet on right ventricular systolic function was independent of the left ventricle ejection fraction. We suggest that the MedDiet regime with the unique anti-inflammatory properties may induce vasodilation and lessen resistance of the pulmonary vascular bed. Such physiology reduces right ventricular afterload and enhances SRV independently of the left ventricular ejection fraction. It is well known that inflammation correlates with diastolic systolic dysfunction, and this may be an explanation for the deterioration from compensated to de-compensated heart failure [24,25,26].

Moreover, in this study, a greater MedDiet score was associated with a higher augmentation index and improved erectile function; while there was a trend with GLPS. These seemingly contradictory findings may be interpreted by the fact that heart failure physiology of the cardiovascular system is fundamentally distinct and that functions of the LV and the peripheral arterial system are interactive (i.e., ventricular/ vascular coupling). Thus, in order to compensate the reduced left ventricular pumping force and consequently the forward pulse wave, peripheral resistance augments to such point that both actions lessen the wave reflection amplification signal. This may be falsely interpreted as reduced arterial stiffness. High adherence to the MedDiet may reduce inflammation, assist vasodilation and help improving peripheral vascular resistance. Such action reassures erectile potency, a nitric oxide phenomenon based on the capacity of the penile arteries to dilate and augment blood flow to the corpus cavernosum. We clinically identified such physiology by the SHIM-5 score, a simple, cost effective and reliable tool that evaluates erectile dysfunction.

Regarding the reflected wave amplification, a higher AIx may be interpreted as the result of improved vascular performance in the periphery that permits first the reflexion of the forward pulsed volume by the falling myocardium and second to such a degree that it finally enhances the pulse signal. Therefore, in such heart failure conditions a higher AIx may be interpreted as a sign of reduced stiffness mainly in the peripheral vascular bed [27]. The fact that pulse wave velocity, typically an index of central arterial stiffness, did not correlate with the MedDiet score further supports such findings. Moreover, such phenomenon may take place independently of the LVEF. Both actions on peripheral physiology and reflected waves may enhance erectile performance, a vital parameter of quality of life in the male population.

Regarding the role of prolactin in heart and vessels diseases, it is increasingly recognized that this hormone is involved in cardiovascular pathophysiology, exerting pleiotropic unfavourable haemodynamic and pro-atherosclerotic actions, raising overall cardiovascular risk [6,9,28,29]. Hyperprolactinaemia has been related to endothelial dysfunction and arterial stiffness [3], low-grade inflammation [2,5], increased thromboembolic risk [7] and dyslipidemia [2]. For example, plasma prolactin levels are elevated in conditions such as the acute phase of myocardial infarction, ischemic stroke, and transient ischemic attack, hypertension and preeclampsia, while evidence suggests a causative role of prolactin in postpartum cardiomyopathy [5,6,7,8,28]. Furthermore, prolactin may accelerate arteriosclerosis in women in early menopause by increasing central as well as peripheral blood pressure and arterial stiffness [9]. Prolactin levels seem to have a prognostic role of future CVD events in men with erectile dysfunction [2] and was associated with all-cause and CVD mortality in the general population, as well as in patients with chronic kidney disease [8]. However, the particular role of prolactin in heart failure in particular has not been fully understood. While small studies have reported elevated serum levels of prolactin in heart failure patients [5], no relationship was detected between prolactin circulating levels and NT-proBNP levels in heart failure elderly patients [29]. No association was also found between CVD mortality and prolactin concentration during 10 years of follow-up [29]. On the contrary, a more recent study indicated that in patients with heart failure of either ischemic or non-ischemic etiology, serum prolactin levels were closely related to functional status and exercise capacity, while acting as an independent prognosticator of total death or hospitalization for cardiac reasons [7].

This study revealed the association between prolactin and IMT, confirming prolactin’s atherogenic properties [30]. However, previous studies yield contradictory results. Subjects with untreated prolactinoma presented increased carotid IMT [31], whereas in premenopausal women high-normal prolactin levels were shown to correlate with lower carotid IMT [32]. In post mortem human coronary arteries obtained after endarterectomy, increased expression of prolactin receptor was found in advanced atherosclerotic arterial walls, whereas no expression was detected in non-significant atherosclerotic lesion, suggesting a modulating role of prolactin in atherosclerosis process [9]. According to our findings, the MedDiet may indeed mitigate the atheromatic effect of prolactin, as high adherence to MedDiet was negatively associated both with IMT and circulating prolactin, suggesting a beneficial atheroprotective effect in patients with systolic heart failure and systolic dysfunction.

Heart failure remains the late serious clinical condition of CVD. As cardiovascular system works in a conduit of the heart and the whole arterial tree, any alterations in cardiac function have impact on arterial properties, and vice versa. The measured central aortic pressure waveform represents the sum of the forward arterial wave, generated by the left ventricle during ejection, and the backward moving wave, caused by the reflection of the forward moving wave within the arterial system. Thus, central aortic pressure waveform expresses the systolic function of the left ventricle, the elastic properties of the entire arterial tree and the interaction between ventricle and aorta, described as aorto-ventricular coupling [33,34]. In the progress of heart failure as left ventricle dilation occurs and the ventricles lose their compensation, the heart muscle cannot generate the necessary extra force to overcome the late systolic augmented pressure. Thus, augmented pressure, systolic and pulse pressure become diminished in parallel with the systolic ejection duration shortening, as the wave reflection poses a negative influence on flow. In this scope, interventions that can modify contractility of the left or right ventricle, can offer a benefit effect on arterial function, improving ventricular-aortic coupling, which will be expressed by an increase in augmentation index and improved clinical status of the patients.

In conclusion, adherence to the MedDiet regime in systolic heart failure male patients posed a significant improvement in cardiovascular physiology including systolic function, parameters of arterial stiffness, atheromatosis and erectile performance. In this particular finding on a male patient population with raised mortality and morbidity MedDiet dietary pattern can be highly recommended as a lifestyle culinary option to maintain cardiovascular health and the sense of wellbeing.

## Figures and Tables

**Table 1:** Demographic and clinical characteristics, ultrasound and exercise test measurements by Mediterranean diet score (MedDietScore) tertile.

Clinical and Biochemical Parameters	MedDietScore Tertiles			*p*-Value
	<28	28–32	≥32	*p*
Age (years)	62.7 ± 8.6	67.3 ± 6.7	64.5 ± 9.9	0.34
BMI (kg/m^2^)	30.0 ± 4.7	27.6 ± 3.4	27.9 ± 3.0	0.19
Diabetes Mellitus (%)	28.6	35.7	16.7	0.55
Hyperlipidemia (%)	55.0	41.2	35.0	0.35
Hypertension (%)	75.2	57.8	73.5	0.53
EF (%)	27.7 ± 6.7	28.9 ± 4.7	26.5 ± 4.7	0.60
METS (per min/day)	6.6 ± 2.1	7.7 ± 2.7	6.3 ± 2.6	0.29
PWV (cm/sec)	9.3 ± 2.3	8.8 ± 1.3	8.5 ± 1.3	0.56
AIx (%)	24.2 ± 7.1	23.1 ± 6.7	22.8 ± 4.3	0.07
IMT (mm)	1.1 ± 0.2	0.8 ± 0.1	0.8 ± 0.1	<0.001
Pulse Pressure (mm/Hg)	36.4 ± 7.2	42.4 ± 11.8	49.1 ± 19.1	0.06
SRV (cm/sec)	8.1 ± 2.0	9.6 ± 2.8	10.1 ± 2.0	0.08
LA volume (ml)	62.6 ± 15.9	66.5 ± 41.4	60.8 ± 14.1	0.88
GLPS (%)	−7.2 ± 2.7	−7.8 ± 4.1	−11.4 ± 5.6	0.10
VO_2_ max (ml/k/min)	18.5 ± 4.5	18.4 ± 3.6	18.4 ± 4.1	0.99
VE/VCO_2_	37.7 ± 7.0	37.0 ± 6.4	36.2 ± 8.1	0.93
Prolactin (ng/dL)	10.3 ± 1.6	20.7 ± 10.4	12.3 ± 3.9	0.05
Testosterone (ng/dL)	389 ± 71	403 ± 74	538 ± 79	0.03
SHIM-5 (range 0–25)	9.1 ± 2.6	12.8 ± 4.0	13.8 ± 6.0	0.006

BMI: Body Mass Index; PWV: Pulse Wave Velocity; AIx: Augmentation index; IMT: Intima Media Thickness; EF: Ejection Fraction; SRV: Systolic Wave of Tricuspid Annulus; LA: Left atrium; GLPS: global longitudinal strain of the left ventricle. *p*-values derived from ANOVA test for the normally distributed variables (age, BMI, EF, PWV, Aix, IMT, Pulse pressure, LA vol, VE/VCO_2_, testosterone), Kruskal-Wallis test for the skewed variables (VO_2_, prolactin, Sexual Health Inventory for Men (SHIM-5)) and chi-square for the categorical variables (hypertension, hyperlipidemia, and diabetes).

**Table 2:** Results from regression models that evaluated the role of Mediterranean diet adherence (through MedDietScore, independent variable) on cardiovascular hemodynamics factors (outcome), in chronic heart failure patients.

Regression Models	Standardized b-Coefficient	*p*-Value
Model for: PWV	−0.073	0.37
Model for: AI x	−0.116	0.014
Model for: IMT (mm)	−0.030	0.004
Model for: Pulse Pressure (mm/Hg)	0.239	0.169
Model for: SRV	0.348	0.019
Model for: LA volume	−0.296	0.590
Model for: GLPS	0.330	0.18
Model for: VO_2_ max	−0.216	0.656
Model for: VE/VCO_2_	0.465	0.431
Model for: SHIM-5	0.617	0.002
Model for: Prolactin (ng/mL)	0.237	0.71
Model for: Testosterone (ng/mL)	0.097	0.59

All models were adjusted for age, BMI, history of diabetes, hyperlipidemia, hypertension, EF and metabolic equivalents (METS).

**Table 3:** Partial correlation coefficients between prolactin, testosterone and cardiac hemodynamic markers, after adjusting for age, BMI, and EF.

	Testosterone	PWV	IMT	PP	SRV	LA Vol	GLPS	VO_2_ max	AIx	SHIM-5
Prolactin	−0.728 *	−0.073	0.070	0.253	0.707 *	0.068	0.190	−0.466 *	0.372 *	−0.689 *
Testosterone		0.170	0.373	−0.394	−0.315	0.226	−0.135	0.290	−0.092	0.342 *
PWV			0.292	0.037	−0.750 *	−0.387	0.126	0.832 *	0.408 *	0.552 *
IMT				−0.820 *	−0.458 *	0.308	0.694 *	0.529 *	0.151	−0.324
PP					0.479 *	−0.452 *	−0.796 *	−0.279	−0.533 *	0.471 *
SRV						0.051	−0.422 *	−0.731 *	−0.094	−0.391
LA vol							0.324	−0.542 *	−0.208	−0.239
GLPS								0.309	−0.665 *	−0.636 *
VO_2_ max									−0.284	0.225
AIx										0.799 *

* *p* < 0.05.

## Data Availability

Data available on request due to restrictions e.g., privacy or ethical. The data presented in this study are available on request from the corresponding author.
